# Derivatization of Lignin via Ternary Eutectic Solvent Systems for Enhanced Functionalities Hydrogel

**DOI:** 10.3390/ma18235283

**Published:** 2025-11-23

**Authors:** Fengfeng Li, Tianci Qin, Xiuxin Yin, Zhili Zhang

**Affiliations:** 1 Jiangsu Provincial Key Lab of Pulp and Paper Science and Technology, Nanjing Forestry University, Nanjing 210037, China; qhxylifeng@163.com; 2 State Key Laboratory of Green Papermaking and Resource Recycling, Qilu University of Technology, Shandong Academy of Sciences, Jinan 250353, China; 15039268772@163.com (T.Q.); Yinxiuxin0920@163.com (X.Y.)

**Keywords:** lignin, derivated lignin, hydrogel, absorption, retention

## Abstract

This study presents a novel structural modification strategy for lignin, utilizing a ternary eutectic solvent system (TESS), which induces targeted derivatization. The resulting lignin-based functional hydrogel (LBFH), prepared via rational cross-linking of derivatized lignin precursors, exhibits exceptional hygroscopic properties, with a water swelling ratio of 934.0%. Water absorption kinetics were subjected to rigorous analysis through the employment of a dual-modeling strategy that incorporates Schott kinetics and Fickian diffusion mechanisms, thereby elucidating the synergistic dynamic processes underlying surface adsorption and matrix penetration. Remarkably, LBFH maintains 48.6% water retention capacity after 7 days atmospheric exposure (25 °C, 60% RH), demonstrating unprecedented environmental stability among biopolymer hydrogels. The engineered properties of LBFH suggest its potential application in sustainable agricultural practices as drought-resistant soil amendments, and in environmental remediation as contaminant-adsorptive matrices.

## 1. Introduction

In recent decades, the pursuit of sustainable materials has become an urgent global imperative [1,2], driven by the escalating concerns over environmental degradation, resource depletion, and the need to transition towards a circular economy [3,4]. Lignin, as one of the most abundant and renewable biopolymers on earth, has emerged as a focal point in materials science research, presenting a plethora of opportunities for value-added applications [5,6,7]. One of the most promising avenues is the development of lignin-based hydrogel (LBH), which combines the inherent advantages of lignin with the tunable properties of hydrogel [8,9]. These hydrogels can be designed to possess a wide range of desirable characteristics, including excellent water absorption and retention capabilities, biocompatibility, and mechanical strength, making them suitable for diverse applications in agriculture [10], environmental remediation [11], biomedical engineering, and more [12,13].

The chemical modification of LBH, especially the precise regulation of functional groups and molecular architecture, exerts a remarkable influence on its water absorption and retention capabilities [14]. The structure of lignin is intricate and highly cross-linked, comprising phenylpropane units adorned with diverse functional groups, such as hydroxyl, methoxy, and carboxyl moieties [15,16]. This elaborate structural framework confers upon lignin distinctive chemical and physical characteristics, yet it concomitantly presents significant impediments in the context of its practical application [17]. Specifically, the limited accessibility and low reactivity of its functional groups hinder its effective integration into polymer networks, such as hydrogels, where robust and homogeneous cross-linking is essential. In order to solve this problem, a variety of improvement methods have been adopted [18]. Amaia et al. enhanced the reactivity of alkaline and organosolv lignin (OSL) through chemical modification: peroxidation of alkaline lignin (AL) and hydroxymethylation of OSL [19]. This approach effectively improved the swelling capacity of LBH. Wang et al. carried out chemical modifications on AL and sodium lignosulfonate using maleic acid. This modification grafted carboxyl groups and carbon-carbon double-bond groups onto the lignin, thereby altering its physicochemical properties [20]. Li et al. achieved the preparation of sulfonated lignin with an elevated sulfonation degree via the chemical modification of lignin, employing formic acid as the modifying agent [21]. Nevertheless, lignin modification methods present environmental issues and are complex in operation. They incur high reagent costs and require harsh reaction conditions, as exemplified by hydroxylation, etherification, and acylation. Owing to factors such as slow reaction velocities, difficulties in purification, and the generation of by-products, the yields of these methods are also rather low [22]. TESS, a ternary eutectic solvent system formed by three interacting components with markedly depressed melting points, offers significant advantages for sustainable lignin modification [23,24]. Choline chloride is often described as the hydrogen bond acceptor. This is because the chloride ion (Cl^−^)-anionic part of the choline chloride salt-acts as a strong acceptor of hydrogen bonds. It forms strong hydrogen bonds with hydrogen bond donors, leading to a significant depression in the melting point of the mixture and forming a eutectic liquid at room temperature. Liu et al. achieved effective preservation of lignin‘s inherent structural characteristics particularly β-O-4 linkages during acidolysis through precise modulation of molar ratios between hydrogen bond acceptor choline chloride and hydrogen bond donors oxalic acid with ethylene glycol in the TESS [25]. The TESS strategy developed in this work offers distinct advantages in sustainability and efficiency over conventional methods such as hydroxylation or acylation. Unlike these earlier approaches our TESS operates under significantly milder conditions and eliminates the need for complex catalysts. This method provides a simpler more environmentally benign and efficient pathway to obtain reactive lignin derivatives. Furthermore, while prior research has largely focused on lignin derivatization itself the transition to functional material fabrication remains unrealized. Our work uniquely leverages the TESS environment to create a reactive lignin derivative specifically designed for developing high-performance hydrogels. This application-oriented approach distinguishes our research from previous studies and establishes a new pathway for lignin valorization. The resulting LBFH fabricated from this precursor demonstrates remarkable properties with ultrahigh water absorption capability reaching 934% attributable to its porous framework abundant hydrophilic groups and optimized molecular configuration. The material maintains exceptional moisture retention stability preserving 48.6% of its initial water content after seven days of ambient exposure. Featuring distinctive pore modulation and hydration management characteristics LBFH emerges as a promising sustainable functional material offering innovative solutions for advanced applications.

## 2. Materials and Methods

### 2.1. Materials

The isolation of organosolv lignin (OSL) was conducted strictly following the methods reported in the previous literature, with pine serving as the starting material [26,27]. Choline chloride (ChCl) were provided by (99.5% purity) Shanghai Macklin Biochemical Technology Co., Ltd. (Shanghai, China) Oxalic acid (OA, 99.5% purity) was purchased from China National Medicine Group Chemical Reagent Co., Ltd. (Beijing, China) Ethylene glycol (EG, 99.5% purity) was achieved from Tianjin Fuyu Fine Chemical Co., Ltd. (Tianjin, China) N,N′-Methylenebisacrylamide (MBA, 99.0% purity), acrylamide (AM, 99.0% purity) and other chemicals were provided by Shanghai Macklin Biochemical Technology Co., Ltd. All reagents were used directly without further purification.

### 2.2. Synthesis of TESS

In the TESS, the molar ratio of ChCl to EG was set at 1:2, and the mass fraction of OA was 0~3 wt% (Table 1). The TESS strategy was selected over traditional acylation or etherification methods due to its ability to achieve efficient lignin derivatization under milder, homogeneous conditions with reduced side reactions. The specific ChCl:EG molar ratio of 1:2 was adopted from the foundational work of Liu et al. [25], as this proportion optimally forms a stable deep eutectic solvent with favorable viscosity and reactivity. The oxalic acid (OA) concentration was limited to 1–3% based on preliminary experiments indicating that this range provides sufficient catalytic activity for derivatization while minimizing acidic degradation of the lignin backbone. These three components were thoroughly mixed. Subsequently, the mixture was heated and stirred at 80 °C with a rotation speed of 300 r/min until a completely transparent solution was formed [28,29]. After that, the solution was hermetically sealed and stored for future use.

### 2.3. Synthesis of Derivated OSL (DOSL)

**Scheme 1:**
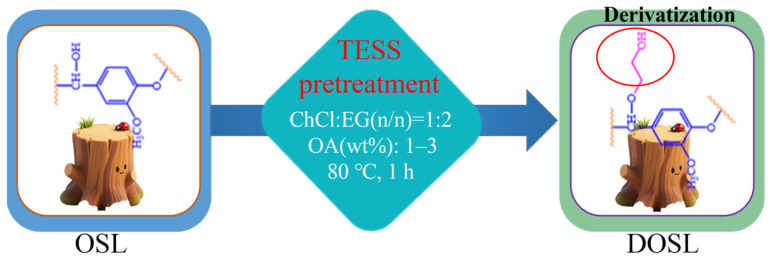
The TESS enables one-step preparation of DOSL from OSL.

The synthesis of DOSL is carried out via a simple one-step method, as shown in Table 2 and Scheme 1. DOSL refers to organosolv lignin that has been chemically modified via grafting with ethylene glycol, forming ether bonds (C–O–C) at the sites of its native hydroxyl groups. This structural change enhances lignin’s hydrophilicity and introduces functional groups facilitating subsequent cross-linking. Firstly, 1.0 g of OSL is added to 5 mL of TESS solvent, and the mixture is subjected to stirring treatment at 80 °C at a rotational speed of 200 r/min for 1 h. Subsequently, 20.0 mL of deionized water is added to terminate the reaction. The obtained solid is then transferred into a dialysis bag with a molecular weight cutoff of 4000 Da. The dialysis process is carried out in deionized water for 48 h. After the dialysis is completed, the solid within the dialysis bag is subjected to freeze-drying under vacuum conditions. Through this series of operations, the DOSL product is successfully obtained. Following the reaction, the TESS was recovered by diluting the mixture with water to precipitate the DOSL, followed by filtration. The aqueous TESS phase was then concentrated via rotary evaporation for reuse, minimizing waste and environmental impact.

### 2.4. Synthesis of LBFH

**Scheme 2:**
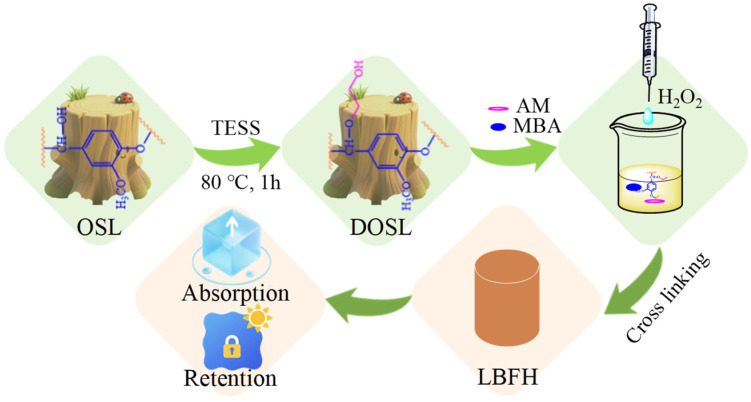
The schematic of the in situ preparation procedure for LBFH.

The preparation of LBFH were carried out using a straightforward in situ method (Table 3 and Scheme 2). Typically, 10–40 g of OSL was added to 5 mL of TESS. The reaction mixture was then stirred at 80 °C and 200 r/min for 1 h. Subsequently, the pH of the reaction system was adjusted to neutrality with a 0.2 M NaOH solution. Then, 0.2 g of AM and 4.0 mg of MBA were added, and the mixture was continuously stirred at 60 °C and 400 r/min until completely dissolved. Finally, 70 μL of 30% H_2_O_2_ was added, and the system was cross-linked at 60 °C for 6 h to obtain the pre-product of LBFH. The pre-product was dialyzed in a 4000 Da dialysis bag for 24 h. This process removed low molecular weight compounds such as unreacted starting materials residual solvents and lignin degradation fragments. After that, it was freeze-dried to obtain the final LBFH products.

### 2.5. Characterization of Samples

The FT-IR spectra of the samples were determined using a Fourier Transform Infrared Spectrometer, with the instrument model (VERTEX 70, Bruker, Bremen, Germany), over a range of 500 to 4000 cm^−1^, at a resolution of 0.5 cm^−1^. The thermal stability of the samples was determined using a synchronous thermal analyzer (TA Instruments, New Castle, DE, USA), with heating from room temperature to 700 °C at a rate of 20 °C/min under a nitrogen protective atmosphere (flow rate: 30 mL/min). Elemental analysis was performed by XPS (ESCALAB Xi+, Thermo Fisher Scientific, Waltham, MA, USA). The sample preparation method provides a suitable sample configuration for XPS analysis, and can accurately determine the elemental composition and chemical state of the elements in OSL and DOSL. Micromorphological analysis was conducted using a field-emission scanning electron microscope (S-5500, Hitachi Hi-Tech, Tokyo, Japan), with a sputter-coated gold layer of approximately 1–2 nm in thickness. Crystallinity of the samples was determined using an X-ray polycrystalline diffractometer (D8 ADVANCE, Bruker, Bremen, Germany) under the following conditions: 20 mA current, 30 kV voltage, Cu target, 2θ range of 10~50°, and scan rate of 0.02°/s. The hydrodynamic diameter of lignin nanoparticles was measured using a Malvern Instruments Zetasizer Nano ZS90 analyzer (Malvern Panalytical, Malvern, UK) with triplicate testing per sample, adopting the optimal result. The molecular weights were determined by GPC (Waters 2695, Milford, MA, USA) using THF at 1.0 mL/min flow rate. Lignin samples underwent acetylation in pyridine-acetic anhydride under dark shaking for 24 h, followed by acid hydrolysis. The treated samples were dissolved in HPLC-grade THF, refrigerated, and analyzed by GPC. The molecular weight was determined from three independent measurements, and the values are reported as the mean.

### 2.6. Water Absorption Swelling and Anti-Dehydratioan Performance

The swelling properties were determined using the gravimetric method, with the following testing steps: At 25 °C, the freeze-dried hydrogel was immersed in deionized water. At regular intervals, it was taken out with tweezers, the surface water was wiped off with filter paper, and then the hydrogel was weighed. Each measurement was performed in triplicate, and the results are presented as the mean value.

WSR (%) = (M_2_ − M_1_)/M_2_ × 100, (1)

where M_1_ represents the completely dry mass of the hydrogel (g) and M_2_ represents the mass of the hydrogel at time t (g).

WLR (%) = (M_0_ − M_d_)/M_0_ ×100, (2)

where M_0_ represents the mass of the fully swollen samples (g) and M_d_ represents the mass of the hydrogel after 1 to 7 days (g). All experiments were performed with at least three independent replicates. Quantitative data are presented as mean values, with error bars representing the standard deviation.

### 2.7. Swelling Kinetics of Hydrogel

The Fickian model is based on Fick’s laws of diffusion, which describe the movement of substances from regions of higher concentration to regions of lower concentration. In the context of hydrogel swelling, water molecules diffuse into the hydrogel network [30]. This model assumes that the hydrogel is a homogeneous medium, and the diffusion of water is the rate-determining step for the swelling process. The driving force for water diffusion is the concentration gradient of water between the external environment and the interior of the hydrogel. The swelling ratio (W) of the hydrogel as a function of time (t) can be obtained by solving the diffusion equation. For the case of a thin-film hydrogel, the relationship between the W and t is given by the following formula [31]:

logW − logW_eq_ = 1/2logt + logK_F_, (3)

where W is the initial swelling ratio, and K_F_ is a constant related to the diffusion coefficient and other parameters. As time progresses, when the hydrogel approaches swelling equilibrium, the swelling ratio W approaches equilibrium swelling ratio (W_eq_).

The Schott model is a semi-empirical model that takes into account both the diffusion of water into the hydrogel and the relaxation of the polymer chains in the hydrogel network during the swelling process. It assumes that the swelling of the hydrogel is a complex process involving both the penetration of water molecules into the network and the rearrangement of the polymer chains to accommodate the absorbed water. The Schott model is expressed as the following formula [31]:

t/W = 1/(K_s_·W_eq_^2^) + 1/W_eq_, (4)

where W represents the swelling rate of hydrogel at time t; W_eq_ is the swelling rate of hydrogel at equilibrium; K_s_ is the characteristic constant. The W_eq_ and K_s_ values can be calculated from the slope and intercept of the fitted curve, respectively.

## 3. Results

### 3.1. Characterization of OSL and DOSL

**Figure 1:**
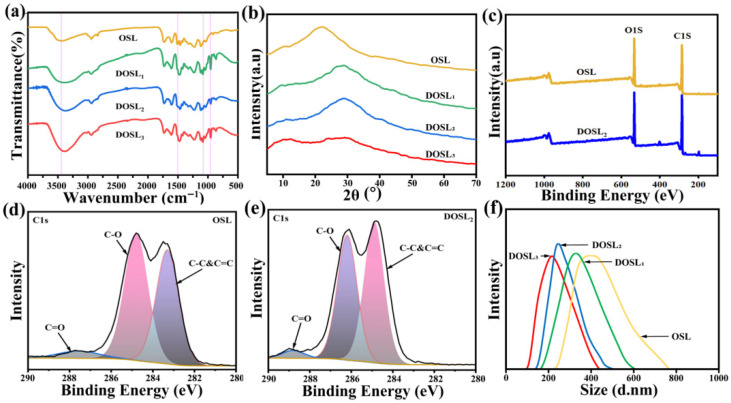
(**a**) FTIR spectra of OSL and DOSL samples; (**b**) XRD patterns of OSL and DOSL_2_ samples; (**c**) XPS analysis and high-resolution spectra of C1s obtained for OSL (**d**) and DOSL_2_ (**e**) samples; (**f**) particle size distribution of OSL and DOSL.

As shown in Figure 1a, spectra in the range of 3000–3500 cm^−1^ correspond to hydroxyl vibrations, while peaks between 1000 and 1300 cm^−1^ represent C-O and C-H bonds [32]. The weakening of the signals at 1315 and 1230 cm^−1^ is consistent with the expected decrease in aromatic C-O stretching vibrations following the grafting reaction with ethylene glycol, though contributions from other factors cannot be ruled out [33]. The signal of lignin on these characteristic absorption peaks was weakened, which demonstrated that the lignin was successfully grafted with ethylene glycol after shrinkage. During the pretreatment process in the TESS, the derivatization pathway of lignin predominates over competing reactions, effectively mitigating potential β-O-4 bond cleavage [25]. The X-ray diffraction patterns of both OSL and DOSL (Figure 1b) display a typical broad scattering halo centered at approximately 2θ = 24°, confirming their amorphous character. No sharp crystalline peaks were detected, as expected for lignin-based polymers [34,35]. The XRD analysis confirmed the expected amorphous morphology of the samples, ensuring that the subsequent functionalization did not alter the fundamental physical state of the lignin backbone. The spectral features corresponding to C-C and C=C bonds demonstrate a clear enhancement in DOSL_2_ (Figure 1c–e). The XPS analysis revealed an increase in the relative abundance of carbon atoms involved in C-O bonds, along with a slight shift in the corresponding binding energy, indicating a change in the chemical environment following the modification process. As depicted in Figure 1f, in comparison with OSL, DOSL exhibits a more favorable distribution concentration and a smaller particle size. This phenomenon can be correlated to the difference in molecular weight between DOSL and OSL. Specifically, DOSL_2_ demonstrates an even more uniform and concentrated particle size distribution. The decrease in molecular weight and particle size unequivocally demonstrates that bond cleavage reactions prevail over condensation pathways in determining the macromolecular properties of DOSL. Although the TESS environment can promote localized condensation, leading to new C-C bonds [25], the extensive acid-catalyzed depolymerization results in a net reduction in size. The spectral features in Figure 1e are thus interpreted as a complex outcome of both bond cleavage and structural rearrangement. As presented in Table 4, when contrasted with OSL samples, DOSL exhibited a lower molecular weight and a more homogeneous particle size distribution. This finding is consistent with the results obtained from the particle size analysis. DOSL_2_ exhibited the highest oxygen content (23.33%), the lowest PDI (1.62), and the smallest particle size among the DOSL samples, indicating a more homogeneous molecular population. Consequently, for the subsequent preparation of LBFH, a system with an OA content of 2 wt% was selected. This choice was based on the favorable characteristics of DOSL_2_, which were expected to contribute to the successful synthesis and performance of the LBFH material.

### 3.2. Characterization of LBFH

**Figure 2:**
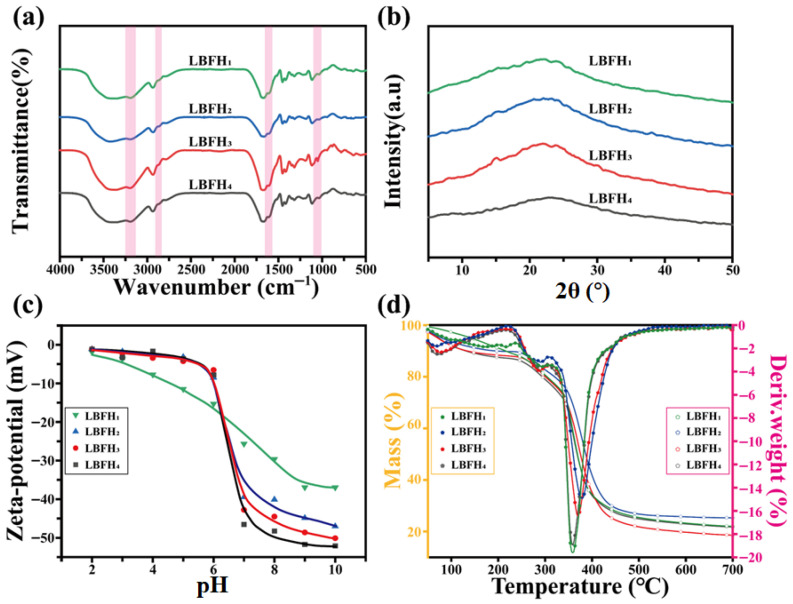
(**a**) FTIR spectra of LBFH samples; (**b**) XRD patterns of LBFH samples; (**c**) TGA and DTG tests of LBFH; (**d**) zeta potential analysis of LBFH.

In Figure 2a, the LBFH exhibits a novel characteristic absorption peak at 3200 cm^−1^. This peak was attributed to the N-H stretching vibration, providing evidence for the successful grafting of AM onto the lignin molecular chain. Furthermore, the characteristic absorption peak at 1660 cm^−1^, originating from the stretching vibration of C=O, further corroborated the successful grafting of AM onto the lignin molecular chain. This dual peak evidence from the infrared spectrum analysis strongly indicates the chemical modification of lignin with AM, which is crucial for understanding the structural transformation and potential applications of the LBFH system [36,37]. As depicted in Figure 2b, LBFH_1–4_ specimens display a broad diffraction peak at 2θ = 24°. Similarly, OSL exhibits a comparable broad diffraction peak at the same 2θ value of 24°. Notably, in both cases, the absence of sharp, characteristic absorption peaks is evident. The XRD analysis reveals a predominantly amorphous structure for the LBFH samples, as evidenced by the broad diffraction peaks and the lack of sharp crystalline reflections.

As can be observed from Figure 2c, with the increase in OSL content, LBFH exhibits a greater absolute value of zeta potential. This indicates that the increment in OSL content enhances the dispersion stability of LBFH in solution. The zeta potential of the hydrogels became increasingly negative with higher OSL content. This trend is attributed to the introduction of acidic functional groups from the OSL, which ionize in aqueous media and contribute negative surface charge. While a more negative zeta potential generally enhances colloidal dispersion, its primary significance in this hydrogel system lies in its correlation with improved adsorption capacity for cationic pollutants, due to strengthened electrostatic interactions. The mechanical properties and cross-linking density of the hydrogel, however, remain the dominant factors governing its overall structural stability and application performance. Figure 2d presents the TGA and DTG curves of LBFH samples. The TGA curve shows a slight mass loss near 100 °C, corresponding to the release of moisture. The predominant and rapid degradation starting at approximately 300 °C is consistent with the decomposition of the lignin backbone, including the cleavage of inter-unit linkages like β-O-4 aryl ether bonds. From the figure, it is evident that the thermal degradation curves of LBFH samples with varying lignin contents are analogous, demonstrating a consistent thermal degradation trend. Figure 2c demonstrates that increasing the lignin content in the hydrogel elevates its initial degradation temperature. A plausible explanation for this phenomenon is that lignin forms chemical linkages with cross linkers and AM, thereby enhancing the thermal stability of the hydrogels [38].

**Figure 3:**
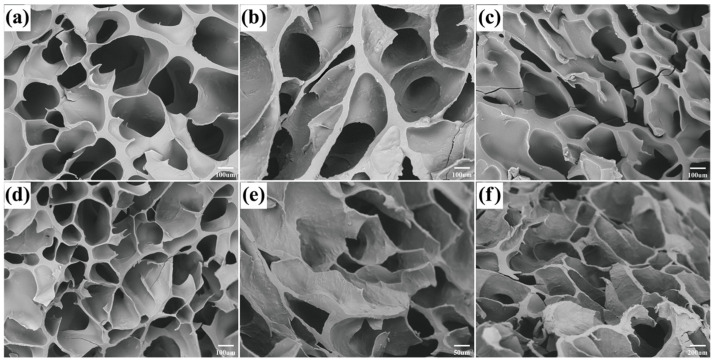
SEM images of LBFH at different magnifications: (**a**) LBFH_1_; (**b**,**e**) LBFH_2_; (**c**,**f**) LBFH_3_; and (**d**) LBFH_4_.

As depicted in Figure 3, the surface structures of LBFH samples with diverse OSL contents were comprehensively characterized. Evidently, the surface of LBFH exhibits an abundance of irregular pores, with dimensions spanning from several to tens of microns. This porous architecture significantly facilitates the infiltration of water molecules, thereby augmenting the water absorption and swelling capacities of LBFH. Notably, a decrease in the pore size of LBFH is observed with the progressive increase in OSL content. While quantitative image analysis was not performed, the SEM micrographs suggest a visual trend of decreasing average pore size. This enhanced interaction modulates the pore size distribution on the hydrogel surface. Moreover, the augmented chain density confers upon LBFH remarkable water retention properties [39].

### 3.3. Characterization of Water Absorption Swelling and Anti-Dehydration Performance

**Figure 4:**
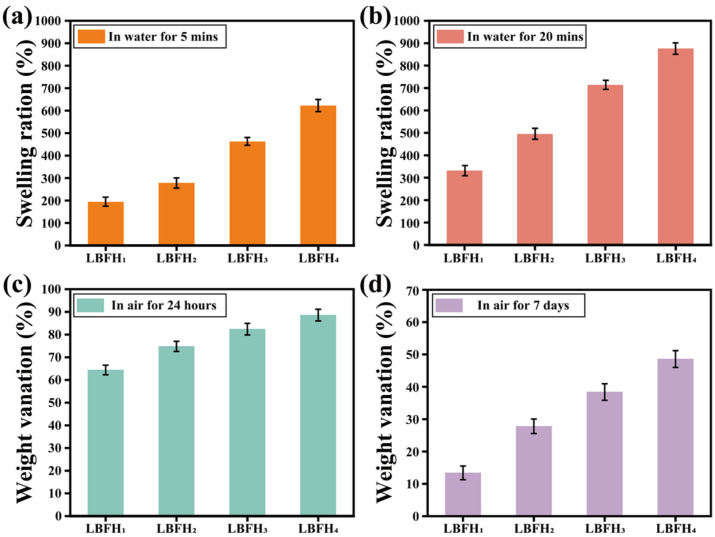
(**a**) Swelling ratio of LBFH after 20 min immersion; (**b**) Swelling ratio of LBFH after 40 min immersion; (**c**) Weight variation in LBFH after 24 h exposure to ambient conditions; (**d**) Weight variation in LBFH after 7 days of exposure to ambient conditions.

The swelling behavior of LBFH was evaluated by determining their swelling ratios in water for 5 min (Figure 4a) and 20 min (Figure 4b). In Figure 4a, for the 5-min immersion, the swelling ratio shows a distinct increasing trend with the sample sequence from LBFH_1_ to LBFH_4_, ascending from ~200% to over 600%. When the immersion duration is extended to 20 min (Figure 4b), all samples exhibit remarkably higher swelling ratios, and the increasing tendency with respect to the LBFH sequence becomes more prominent-LBFH_4_ achieves a swelling ratio of ~850%, nearly fourfold that of LBFH_1_ (~300%). The swelling and water retention properties of the LBFHs are intrinsically governed by their network architecture, which varies systematically with lignin content. LBFH_1_, with the lowest lignin ratio, demonstrates higher swelling capacity, attributable to its more flexible and hydrophilic polyacrylamide-dominated matrix. In contrast, LBFH_4_, with the highest lignin incorporation, shows more restrained swelling, resulting from a denser network structure with increased hydrophobic aromatic segments and physical cross-links. This clear structure-property relationship highlights the role of lignin content in tuning the hydrogel’s interaction with water, enabling the rational design of materials with tailored hydration behavior for specific applications. When LBFH comes into contact with water, the hydrophilic groups within the hydrogel, such as those associated with the AM component, attract water molecules through hydrogen bonding and other intermolecular forces [40]. The pores on the surface of LBFH, ranging in size from several to tens of microns, provide channels for the ingress of water molecules. With an appropriate increase in OSL content, the enhanced interaction between OSL and AM may optimize the pore size distribution, potentially increasing the water absorption rate. A uniformly distributed pore structure is conducive to more efficient water penetration. As observed from Figure 4c,d when LBFH samples are placed in air for one day, the water retention effect improves progressively with the gradual increase in OSL content. Notably, for LBFH_4_, the water loss resistance rate in air reaches 48.6% (Figure 4d). This retention level is attributed to the incorporation of lignin, which contributes to a densely cross-linked network with hydrophobic segments that effectively slow water evaporation. The relationship between lignin content and water retention was clearly observed across the LBFH series, with higher lignin levels corresponding to improved long-term moisture preservation. While the complete drying kinetics were not characterized, the data at these key intervals effectively demonstrate the superior long-term hydration retention of the LBFHs, particularly those with higher OSL content. These results demonstrate the potential of lignin as a key component in designing hydrogels with enhanced environmental stability. This is evident from the SEM results (Figure 3), which show a noticeable shift in surface porosity and pore architecture. The smaller pore size establishes a physical barrier that effectively slows down the rate of water evaporation. Specifically, the diminished pore dimensions limit the mobility of water molecules, rendering it more arduous for them to escape from the material’s interior to the external environment. Moreover, with the growth in OSL content, the chain density within the system is elevated. In essence, even under relatively dry external conditions, LBFH can uphold a high water content and demonstrate excellent water retention capabilities, attributable to the distinctive structure engendered by the increase in lignin content.

### 3.4. Water Absorption and Swelling Models

**Figure 5:**
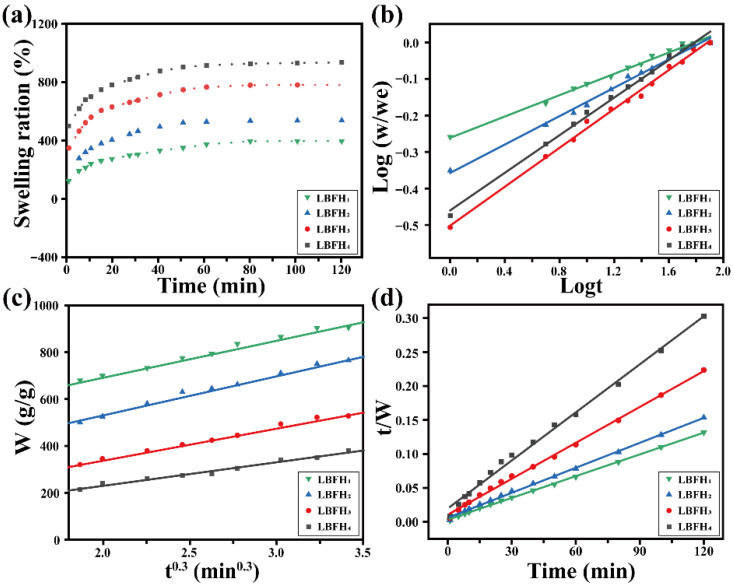
(**a**) Swelling rates of LBFH in deionized water at 25 °C; (**b**) Plots of log (W/We) against logt for LBFH; (**c**) The LBFH fits Fickian model curves within the range of 5~45 min at 25 °C; (**d**) The curves were fitted by LBFH Schott model at 25 °C.

The water absorption and swelling characteristics of LBFH were meticulously investigated using the gravimetric approach. As depicted in Figure 5a, the swelling rates of all hydrogels manifest two distinct phases. In the initial phase, the swelling rate escalates precipitously; in the subsequent phase, it stabilizes and remains essentially constant. All four hydrogels reached swelling equilibrium at approximately 60 min, with equilibrium swelling ratios of 934%, 781%, 537%, and 396%, respectively.

Lignin generally exhibits a certain degree of hydrophobicity, exerting a unique impact on the water absorption properties within composite materials. The pore size of LBFH diminishes with the increase in OSL content. This can be primarily attributed to the fact that as the lignin content rises, the interaction between OSL and AM intensifies, thereby altering the pore size distribution on the hydrogel surface. The reduced pore size is disadvantageous for the influx of a large number of water molecules, restricting the water absorption rate and capacity of the hydrogel to a certain degree.

Simultaneously, the inherent hydrophobicity of lignin gives rise to a relatively hydrophobic microenvironment in its vicinity, further impeding the infiltration of water molecules. Additionally, higher lignin content increases the chain density of the network, forming a more tortuous structure that restricts water molecule diffusion and reduces evaporative water loss, thereby improving water retention performance. As presented in Table 5 and Figure 5c,d the swelling kinetics of the LBFHs were analyzed using both the Fickian and Schott models. The *n* values derived from the Fickian model fell within the range of 0.25–0.3, suggesting a diffusion-controlled mechanism with a relatively slow water molecule diffusion rate. Concurrently, the Schott model also yielded high linear correlation coefficients (R^2^ ≈ 0.99), indicating a strong fit to the experimental data [41]. The close agreement between the measured and fitted equilibrium swelling rates further supports the applicability of the Schott model [42]. The high goodness-of-fit for both models suggests that the swelling kinetics may not be governed by a single mechanism but likely involve a combination of diffusion and polymer relaxation processes, indicating complex water uptake behavior in the LBFHs. 

## 4. Discussion

The structural evolution of lignin from OSL to DOSL, elucidated by FTIR, XRD, and molecular weight analyses, underscores the success of derivatization. In the FTIR spectra, the attenuation of lignin-specific characteristic peaks, coupled with alterations in C-O bond strength, indicates effective suppression of β-O-4 bond cleavage during the TESS treatment. The reduced molecular weight, narrower PDI, and more uniform particle size distribution of DOSL-alongside its elevated oxygen content-reflect a controlled modification process. For LBFH, the emergence of a characteristic absorption peak at 3200 cm^−1^ in the FTIR spectrum confirms the grafting of AM onto the lignin backbone via N-H stretching vibrations, while the amide carbonyl vibration at 1680 cm^−1^ further validates this chemical modification. The broad XRD peaks of LBFH, devoid of sharp crystalline features, indicate an amorphous or semi-amorphous structure. This structural motif is advantageous: amorphous materials typically exhibit superior dispersibility, increased surface area, and enhanced compatibility in composite systems-properties pivotal for applications in adsorption, catalysis, or functional materials. The porous architecture of LBFHs, as revealed by scanning electron microscopy, exhibits a distinct dependence on the content of OSL, with profound implications for water interaction behaviors. The abundance of irregular pore establishes extensive surface area and interconnected pathways, facilitating efficient water infiltration and subsequent swelling. As OSL content increases, a notable reduction in pore size emerges, attributable to enhanced interactions between OSL and AM. This structural evolution highlights the potential to tailor OSL content for customizing porous properties, enabling applications requiring precise water management. However, deeper investigations into the dynamic interplay between OSL, AM, and water during swelling are needed to fully unravel the mechanistic basis of these structure–property relationships.

The water absorption and anti-dehydration behaviors of LBFHs reveal a complex interplay between chemical interactions and porous architecture. The rapid achievement of swelling equilibrium within 40 min reflects the synergistic effects of the hydrophilic groups in the AM component and the interconnected porous network. Hydrogen bonding and other intermolecular forces between AM and water molecules facilitate initial water absorption, while the uniformly distributed pores, optimized by the enhanced interaction between OSL and AM, promote efficient water penetration.

Regarding anti-dehydration performance, the gradual enhancement with increasing OSL content stems from two structural changes. The decrease in pore size forms a physical barrier, restricting the mobility of water molecules and reducing evaporation rates. Meanwhile, the increased chain density due to strengthened OSL-AM interactions creates a compact, interwoven network that traps water in its interstitial spaces. This structural reinforcement not only improves the mechanical integrity of the hydrogel but also maintains high water retention even under relatively dry ambient conditions.

The swelling behavior of LBFHs, characterized by a two-phase kinetic profile, reflects the dynamic response of their polymer network to water ingress. The initial rapid swelling phase signifies efficient water penetration driven by the hydrophilic domains within the network, while the subsequent plateau indicates the establishment of a swollen equilibrium, constrained by the network’s elastic resistance. The incorporation of OSL introduces a dual-functional effect on water interaction: on one hand, the enhanced OSL-AM interactions reduce pore size and increase chain density, creating a more compact network that impedes rapid water infiltration, as evidenced by the decreasing equilibrium swelling ratios with higher OSL content. On the other hand, this structural densification fortifies the hydrogel’s physical barrier, retarding water evaporation and improving retention capacity, a phenomenon consistent with the inherent hydrophobicity of lignin that moderates the hydrogel’s wettability. The diffusion coefficient (*n*) of water molecules in LBFH, ranging between 0.25 and 0.3, aligns with Fickian diffusion behavior, indicating a relatively slow diffusion rate constrained by the narrowed pore structure. The excellent fitting of swelling kinetics to the Schott model, as manifested by correlation coefficients approaching 0.99, validates the model’s applicability and underscores the dominant role of network structure in governing swelling dynamics. This consistency between experimental observations and theoretical modeling elucidates the mechanistic link between OSL-mediated structural evolution and water-polymer interactions.

The LBFHs exhibits significant potential for applications in sustainable agriculture and eco-friendly remediation, owing to its high water retention capacity, controlled swelling behavior, and adsorptive functionality. When compared to commercial synthetic hydrogels, LBFHs offer a notable advantage in material sustainability and environmental compatibility, as they are derived largely from renewable lignin and avoid persistent synthetic polymer residues. Among biopolymer-based hydrogels documented in research, LBFHs demonstrate superior long-term moisture preservation under ambient conditions, addressing a common limitation of rapid dehydration seen in many bio-based materials. This enhanced performance is attributed to its lignin-enhanced network, which moderates water release and improves atmospheric stability. Further development will focus on optimizing composition for specific use cases and assessing scalability to bridge laboratory findings with practical implementation.

## 5. Conclusions

The strategic implementation of TESS methodology enabled precise lignin modification, effectively mitigating steric constraints while enhancing functional group density through targeted molecular engineering. Comprehensive characterization of LBFH revealed exceptional hygroscopic performance, achieving a 934% equilibrium swelling ratio attributable to its hierarchically porous architecture, hydrophilic domain optimization, and macromolecular design. Notably, LBFH maintained 48.6% water retention after 7 days ambient exposure (25 °C, 60% RH), demonstrating robust dehydration resistance via stabilized hydrogen-bonding networks and capillary confinement mechanisms. These synergistic properties establish LBFH as a dual-functional hydromodulator capable of rapid moisture capture and sustained hydrological release. This study presents a method to valorize lignocellulosic waste into a multifunctional LBFH. The material’s properties suggest it could be further investigated for use in addressing water management challenges in fields such as sustainable agriculture and ecosystem restoration. Future work will explore the practical application potential of the LBFH, particularly in areas such as controlled drug delivery systems and the adsorption of environmental pollutants from aqueous solutions. Investigations into the release kinetics of model drugs and the adsorption capacity for specific contaminants will be conducted to evaluate its efficacy in these fields.

## Figures and Tables

**Table 1:** Synthetic formulations of TESS samples.

Samples ID	ChCl:EG (*n*/*n*)	OA (wt%)	Temperature (°C)	Rotation Speed (r/min)
TESS1	1:2	1	80	300
TESS2	1:2	2	80	300
TESS3	1:2	3	80	300

**Table 2:** Synthetic formulations of DOSL samples.

Samples ID	OSL (g)	TESS	Temperature (°C)	Time (h)	Yield (%)
DOSL_1_	1.0	TESS1	80	1	48.42
DOSL_2_	1.0	TESS2	80	1	53.57
DOSL_3_	1.0	TESS3	80	1	52.01

**Table 3:** Synthetic formulations of LBFH samples.

Samples ID	OSL (g)	TESS2 (mL)	AM (g)	MBA (mg)	Temperature (°C)	Time (h)	Yield (%)
LBFH_1_	10	5	0.2	4.0	60	6	82.42
LBFH_2_	20	5	0.2	4.0	60	6	83.57
LBFH_3_	30	5	0.2	4.0	60	6	86.01
LBFH_4_	40	5	0.2	4.0	60	6	85.97

**Table 4:** Element content and the molecular weight of OSL and DOSL samples.

Samples ID	C (%)	H (%)	O (%)	M_w_ (g/mol)	M_n_ (g/mol)	PDI (M_w_/M_n_)
OSL	78.06	6.12	15.82	5818	2806	2.07
DOSL1	74.24	7.02	18.74	3740	2108	1.77
DOSL2	70.06	6.61	23.33	2692	1660	1.62
DOSL3	71.26	6.62	22.12	2162	1466	1.47

**Table 5:** Correlation coefficients of the Fickian and Schott model.

Samples ID	Fickian Model			Schott Model		
	n	k	R^2^	W_e,cal_ (g/g)	W_e,exp_ (g/g)	R^2^
LBFH_1_	0.2822	0.4628	0.9942	934.24	914.56	0.9943
LBFH_2_	0.2654	0.3648	0.9838	781.25	764.24	0.9976
LBFH_3_	0.2852	0.2642	0.9876	536.82	524.64	0.9984
LBFH_4_	0.2904	0.2840	0.9928	395.97	388.42	0.9992

## Data Availability

The original contributions presented in this study are included in the article. Further inquiries can be directed to the corresponding author.
